# A Novel Multi-Scale Particle Morphology Descriptor with the Application of SPHERICAL Harmonics

**DOI:** 10.3390/ma13153286

**Published:** 2020-07-23

**Authors:** Wei Xiong, Jianfeng Wang, Zhuang Cheng

**Affiliations:** 1 Department of Architecture and Civil Engineering, City University of Hong Kong, Hong Kong, China; Xiong.Wei@my.cityu.edu.hk (W.X.); zhuacheng2-c@my.cityu.edu.hk (Z.C.); 2 Shenzhen Research Institute of City University of Hong Kong, Shenzhen 518000, China

**Keywords:** multi-scale morphology descriptor, spherical harmonic analysis, incremental morphology variation, inter-scale effect, surface roughness heterogeneity, X-ray micro-tomography

## Abstract

Particle morphology is of great significance to the grain- and macro-scale behaviors of granular soils. Most existing traditional morphology descriptors have three perennial limitations, i.e., dissensus of definition, inter-scale effect, and surface roughness heterogeneity, which limit the accurate representation of particle morphology. The inter-scale effect refers to the inaccurate representation of the morphological features at the target relative length scale (RLS, i.e., length scale with respective to particle size) caused by the inclusion of additional morphological details existing at other RLS. To effectively eliminate the inter-scale effect and reflect surface roughness heterogeneity, a novel spherical harmonic-based multi-scale morphology descriptor *R_inc_* is proposed to depict the incremental morphology variation (IMV) at different RLS. The following conclusions were drawn: (1) the IMV at each RLS decreases with decreasing RLS while the corresponding particle surface is, in general, getting rougher; (2) artificial neural network (ANN)-based mean impact values (MIVs) of *R_inc_* at different RLS are calculated and the results prove the effective elimination of inter-scale effects by using *R_inc_*; (3) *R_inc_* shows a positive correlation with the rate of increase of surface area *R_SA_* at all RLS; (4) *R_inc_* can be utilized to quantify the irregularity and roughness; (5) the surface morphology of a given particle shows different morphology variation in different sections, as well as different variation trends at different RLS. With the capability of eliminating the existing limitations of traditional morphology descriptors, the novel multi-scale descriptor proposed in this paper is very suitable for acting as a morphological gene to represent the multi-scale feature of particle morphology.

## 1. Introduction

Particle morphology, an intrinsic characteristic for granular soils, plays a significant role in the grain-scale and, consequently, macro-scale mechanical behaviors of granular soils. Many research findings, either experimental or numerical, proved that the fabric evolution and fabric anisotropy in grain-scale, as well as the compressibility, crushability, shear strength, dilatancy and small strain stiffness in macro-scale, can be highly influenced by the morphology features of granular particles [1,2,3,4,5,6,7,8]. Therefore, the precise and quantitative representation of particle morphology is the prerequisite of further geological and geo-mechanical investigations of granular soils.

To estimate particle morphology like sphericity and roundness, researchers, in early years, compared the microscopic view of particles with the standard charts developed by Krumbein and Sloss [9]. Although quite convenient for a small number of particles, this chart comparing method is very subjective and is not suitable for a population of particles. In recent years, the development of optical equipment and imaging techniques boosted the accuracy and efficiency of representation of particle morphology. For instance, Cho et al. [3] utilized a stereomicroscope to investigate the effects of particle shape on packing density, as well as the mechanical properties of sandy soils. Altuhafi and COOP [10] adopted the QicPic apparatus to correlate soil behavior to the particle microscopic morphology variation. Zheng and Hryciw [11] proposed stereophotography to obtain three-dimensional (3D) surfaces of 600 randomly selected soil particles. Zhao and Wang [12] introduced a framework to quantify the 3D morphology of Leighton Buzzard sand (LBS) particles based on X-ray micro-tomography (*μ*CT). To quantitatively represent particle morphology in a smaller relative length scale (RLS), many advanced mathematical methods were developed, such as the Fourier series [13,14], the fractal dimension [15,16], wavelet analysis [17], and spherical harmonic analysis (SHA) [18,19,20].

Due to the multi-scale nature of particle morphology, the quantitative representation should be conducted in different RLS. In general, particle morphology can be divided by RLS into general form (GF), local roundness (LR), and surface texture (ST) (or roughness in a smaller RLS). Many descriptors were proposed to describe particle morphology to some specific RLS. Although yielding a lot of interesting findings, the limitations of these traditional descriptors are obvious and are listed below.

The first is dissensus of definition. There still remains the dissensus of definitions for the descriptors, leading to an inconsistent set of descriptors utilized by different researchers [21,22,23]. This makes the representation of particle morphology hard to unify and compare. To address this issue, Blott and Pye [21] re-examined the basic concepts of particle shape and suggested descriptors for accurate shape representation. The second is the inter-scale effect. The estimation of morphology descriptors is actually based on the “observation length scale”, which that means techniques with different resolutions will yield different values for the same particle surface [3,23,24]. That is, the inter-scale effects from smaller RLS will lead to an incorrect representation of particle morphology at the target RLS. For instance, the local roundness of a given particle might be significantly underestimated if the surface texture is not appropriately removed, as the surface texture will lower the evaluation of local curvature for a surface point. To avoid the inter-scale effect from smaller RLS, Zhao and Wang [12] proposed a cut-off method to remove morphology information of surface texture, while Zheng and Hryciw [23] adopted a combined locally weighted regression smoothing (LOESS) and K-fold cross-validation method to remove the roughness details and obtain the mean particle surface. These methods can effectively eliminate the inter-scale effect of smaller RLS, but that of large RLS still exists. Moreover, the extension of these methods to other RLS, especially smaller RLS, is hard to conduct. The third is surface roughness heterogeneity. The formation history (such as particle breakage, chipping, and abrasion subjected to loads or transportation) and the mineralogical composition of granular materials lead to different surface roughness at different locations. Unlike the shearing of rock joint surfaces, the inter-particle contact among granular particles occurs in very small areas, which makes the effect of this heterogeneity more obvious and dominant. These three perennial issues widely exist, and they limit the accurate representation of particle morphology at different RLS. 

This paper proposes a novel spherical harmonic (SH)-based multi-scale morphology descriptor that could effectively eliminate inter-scale effects with a uniform format of definition across all RLS and that could reflect surface roughness heterogeneity. Section 2 presents a combined X-ray *μ*CT and SHA technique to acquire morphological data of 4155 LBS particles and then to decompose each particle surface into a series of sub-surfaces at different RLS. Section 3 discusses the effect of SH decomposition and the inter-scale effect on particle morphology representation by selected traditional descriptors. Section 4 then introduces the multi-scale morphology descriptor proposed and discusses its characteristics and advantages. Particularly, an artificial neural network is introduced to correlate the multi-scale morphology descriptor with the SH-based invariants. Finally, Section 5 presents the conclusion remarks.

## 2. Morphological Data Acquisition and SH-Based Particle Surface Decomposition

### 2.1. Data Acquisition by X-ray μCT

X-ray *μ*CT as a powerful tool to visualize and characterize the grain-scale mechanical behavior of granular soils was widely adopted in many researches, including grain morphology [12,22,25], grain-scale kinematics [26,27,28,29], and fabric evolution [30,31]. Due to its three-dimensional, high-resolution, and non-destructive merits, X-ray *μ*CT is utilized in this paper to acquire data of particle morphology. Specifically, LBS with a diameter range of 400–800 μm are utilized to form a dry cylindrical sample with a diameter and a height of 8 mm and 16 mm, respectively. Then, the sample is scanned within a synchrotron-based *μ*CT scanner at the BL13W beamline of the Shanghai Synchrotron Radiation Facility (SSRF). An X-ray energy of 25 KeV and voxel size of 6.5 *μ*m are used for the CT scanning, since this setting can provide a good contrast between solid particles and void spaces [31].

**Figure 1:**
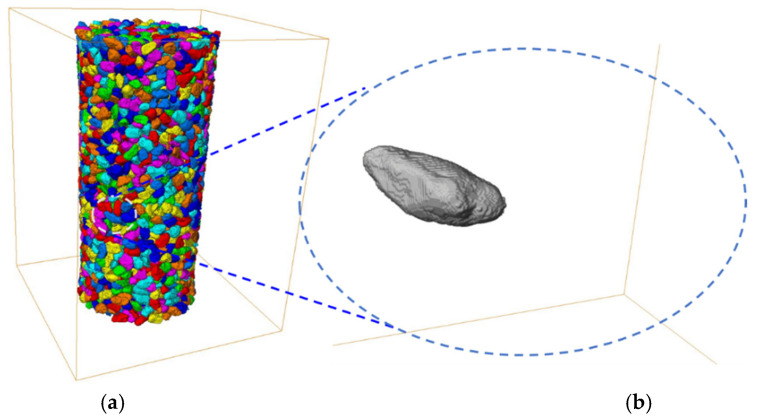
Image obtained from micro-tomography (*μ*CT) reconstruction: (**a**) three-dimensional (3D) labeled image; (**b**) an individual particle.

Once a raw CT image of the sample is acquired, a series of image processing steps and analyses are generally required to extract the surface of each particle. The image processing procedure, in general, is carried out in five steps. Firstly, a series of raw projection images obtained from synchrotron *μ*CT scans at different rotation angles is transformed to gray-scale CT slices on free software PITRE [32] from SSRF [33]. Secondly, an anisotropic diffusion filter method [34] is conducted on those gray-scale CT slices obtained to remove unexpected noise. This filter technique can maintain the original boundaries and enhance the contrast among different phases while dramatically eliminating the noise in the background. Thirdly, an intensity value threshold [35] is utilized on filtered CT slices to convert them into binary images. Fourthly, a marker-based watershed algorithm is adopted on binary images to extract individual particles and store them in a three-dimensional labeled image (Figure 1a). Lastly, the intrinsic MATLAB function *bwprim* is applied to extract particle boundary voxels for each particle in the LBS sample (Figure 1b), while *regionprops3* is utilized to calculate volume and surface area of each particle. A more detailed description of the experimental device and the image processing can be found in Cheng and Wang [31], in which the sample data were used to study the fabric evolution of granular soils under shear.

In order to eliminate the over-segmentation effect, particles with average values of the major and the minor principal axis lengths less than 0.4 mm are removed, and the remaining 4155 LBS particles are extracted for further analysis.

### 2.2. Spherical Harmonic-Based Particle Surface Decomposition

Based on the extracted CT data of individual particles, the particle surface can be decomposed by SHA into a series of sub-surfaces at different RLS. Here, we briefly introduce the SHA procedure for particle surface decomposition. A more detailed description can be found in Zhou et al. [20]. By adopting SH functions, the polar radius of a unit sphere can be extended in different frequencies to match the particle surface points, with different frequencies relating to different RLS. The main functions are shown as follows:

(1) $$r(\theta ,\varphi )\;=\;\sum_{n=0}^{\infty }\sum_{m=-n}^{n}C_{n}^{m}Y_{n}^{m}(\theta ,\varphi ),$$

where r(θ,φ) is the polar radius from the particle center, $C_{n}^{m}$ is the spherical harmonic coefficient, and $Y_{n}^{m}(\theta ,\varphi )$ is the spherical harmonic function as given by Equation (2).

(2) $$Y_{n}^{m}(\theta ,\varphi )\;=\;\sqrt{\frac{(2n+1)(n-m)!}{4\pi (n+m)}}P_{n}^{m}(cos\theta )e^{-i\omega \varphi },$$

where n and m are the frequency and the order of the associated Legendre function $P_{n}^{m}(x)$,

(3) $$P_{n}^{m}(x)\;=\frac{{\left(-1\right)}^{m}}{2^{n}n!}\;{\left(1-x^{2}\right)}^{m/2}\frac{d^{n+m}}{dx^{n+m}}{\left(x^{2}-1\right)}^{n}.$$

**Figure 2:**
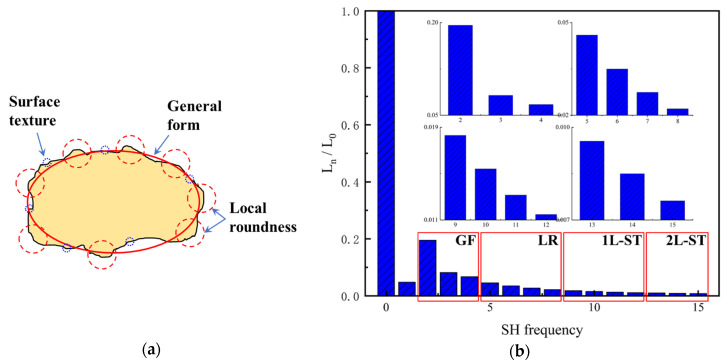
Particle morphology at different relative length scale (RLS): (**a**) schematic diagram; (**b**) averaged second-order norms for each spherical harmonic (SH) frequency.

Based on the spherical harmonic coefficient $C_{n}^{m}$, the given particle surface can be reconstructed using Equation (1). In addition, the second-order norm of these coefficients expressed by Equation (4) reflects the energy contained in each frequency [36] and, hence, can be utilized to partition and group RLS range.

(4) $$L_{n}={\left\|C_{n}^{m}\right\|}_{2}=\sqrt{\sum_{-n}^{n}\left(C_{n}^{m}\times C_{n}^{m*}\right)},$$

where ${\left\|*\right\|}_{2}$ is the second-order norm, and * is the conjugate transpose. Note that *L_n_* is an SH invariant with respect to particle translation and rotation. Since *L_0_* is related to particle size, all the second-order norms are divided by *L_0_* to eliminate the particle size effect. *L_1_* is only related to particle shift and, hence, ignored in the classification. In addition, SHA was already proven to accurately describe the morphology information of LR and ST when the maximum SH frequency is 15 or greater [22,37,38,39] and, hence, SH frequency *n* = 15 is chosen in this paper for further analysis. Figure 2 depicts the multi-scale features of particle morphology and the averaged second-order norms for all the 4155 particles in each frequency. Based on different *L_n_/L_0_* values in each frequency, particle surface can be decomposed into a series of sub-surfaces containing morphological genes at three different RLS, i.e., general form (GF) at *n* = 2 to 4, local roundness (LR) at *n* = 5 to 8, and surface texture (ST) *n* = 9 to 15 [23,39,40,41]. In order to discuss the inter-scale effect at finer RLS, ST is further divided into first-level surface texture (1L-ST) *n* = 9 to 12 and second-level surface texture (2L-ST) *n* = 13 to 15. Therefore, with $C_{n}^{m}$ calculated and Equation (1), any given particle surface can be decomposed into a series of sub-surfaces at different RLS. For instance, the corresponding LR-level sub-surface can be reconstructed by SH frequency n=8 with the first 81 coefficients of $C_{n}^{m}$.

### 2.3. Verification

**Figure 3:**
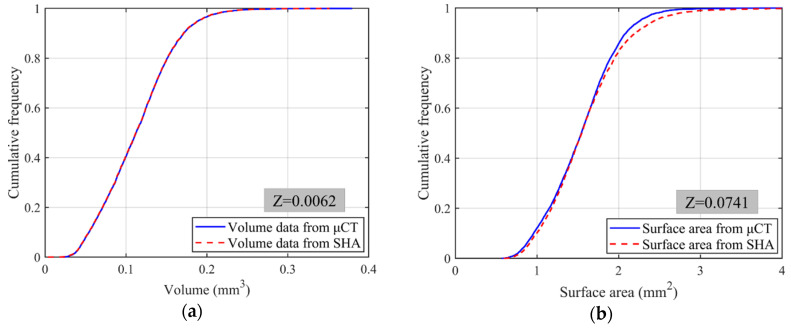
Comparisons between cumulative distributions of 4155 Leighton Buzzard sand (LBS) particles from *μ*CT and spherical harmonic analysis (SHA): (**a**) particle volume; (**b**) particle surface area.

The precise SH reconstruction of particle surface from *μ*CT data is the premise of spherical harmonic-based decomposition. For the verification, particle volumes and surface areas obtained by *μ*CT and SHA are compared using the *Z* value (Equation (5) in a statistical approach, *t*-test [42]. In detail, the calculation of particle volume and surface area for *μ*CT data is conducted using MATLAB functions *regionprops3* and *isosurface*, while these values for SHA data are evaluated using the sum of the micro-surface areas of all faces and the sum of the micro-volumes of all the tetrahedrons, respectively (detailed calculation procedure given by Zhou et al. [20]). The *Z* value, as shown in Equation (5), is related to means (*μ*) and standard deviations (*σ*) of two distributions, and it can be utilized to evaluate the divergence of SHA reconstruction from *μ*CT data.

(5) $$Z\;=\;\frac{\left|\mu_{1}-\mu_{2}\right|}{\sqrt{\sigma_{1}^{2}+\sigma_{2}^{2}}},$$

where *μ_1_* and *μ_2_* are the means of two distributions, while *σ_1_* and *σ_2_* are the standard deviations. For all 4155 LBS particles, the mean and standard deviation of particle volume from *μ*CT data are 0.1136 and 0.0454, while those from SHA are 0.1132 and 0.0453, respectively. Similarly, the mean and standard deviation of particle surface area are 1.5419 and 0.4375 for *μ*CT data, and 1.5923 and 0.5203 for SHA. Figure 3 compares their cumulative frequency distributions from *μ*CT and SHA. Solid lines show the volume and surface area distributions from *μ*CT scans, while dotted lines are from SHA. The Z value is 0.0062 for particle volume and 0.0741 for particle surface area, and they are all within 1.96, which reflects a sufficiently close match between these two distributions with a confidence level of 95% [43]. 

## 3. Inter-Scale Effect of Traditional Morphology Descriptors

The traditional morphology descriptors listed in Table 1 are selected at different target RLS to illustrate their sensitivity to the SH decomposition of particle surface and to further discuss their inter-scale effects. The definitions of these descriptors are based on the recommendations from Blott and Pye [21]. As mentioned above, most existing particle morphology descriptors can be classified by the target RLS into four different groups, namely, GF, LR, ST, and overall shape parameters. To be more exact, (a) GF, such as aspect ratio, is related to the three principle dimensions of a granular particle, (b) LR is considered to be a measure of the sharpness of corners and edges for a particle, (c) average texture (AT) is the averaged absolute distance of surface points to the mean surface, which can show the morphology variation of ST in a very general way, and (d) overall shape parameters are those that can be influenced independently by features at different RLS.

**Figure 4:**
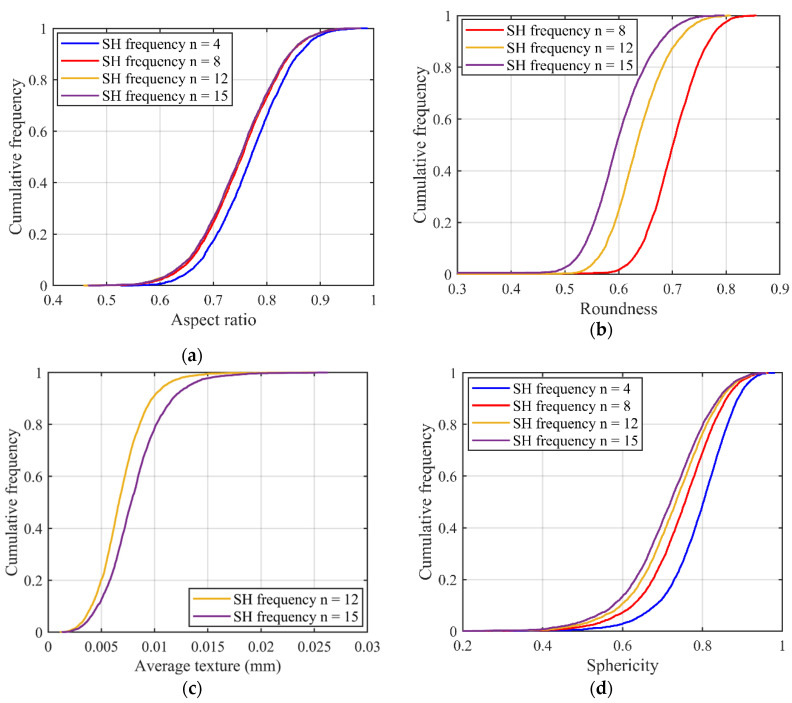

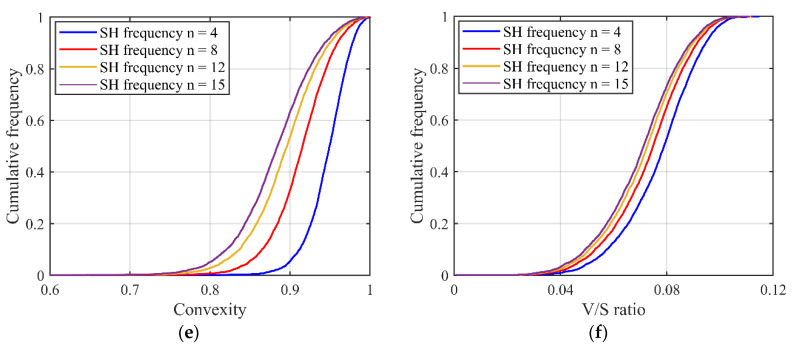
Variation of particle morphology descriptors with SH frequency: (**a**) aspect ratio; (**b**) roundness; (**c**) average texture; (**d**) sphericity; (**e**) convexity; (**f**) V/S ratio.

Based on SHA, particle surface is decomposed into a series of sub-surfaces at different RLS. Then, the chosen traditional morphology descriptors are calculated on each sub-surface and this procedure is repeated for all the 4155 LBS particles. Figure 4 shows the variation of particle morphology descriptors with increasing SH frequency. It is found that all the descriptors show a similar trend of decreasing values with an increasing SH frequency except the average texture, which has an opposite trend. This result shows that the “observation length scale” has a significant effect on the estimation of traditional morphology descriptors, proving the existence of the inter-scale effect. 

However, a big difference in the degree of variation exists in different descriptors, with the largest and smallest variations seen in roundness and V/S ratio, respectively. To be more exact, the aspect ratio (Figure 4a) can be affected by LR-level morphology information, and it shows little sensitivity to high-level morphologies like ST. The LR, from Figure 4b, is highly sensitive to the morphology variation of 1L-ST and 2L-ST and shows a decreasing trend with an increasing SH frequency. The average texture for the ST-level morphology information, as shown in Figure 4c, is sensitive to the smaller RLS such as 2L-ST and witnesses a positive correlation with the SH frequency. The remaining three overall shape parameters (Figure 4d,e,f) reflect the general variation of particle shape. Thus, morphology information from all the different RLS can affect the estimation of these descriptors. Therefore, the inter-scale effect exists in particle morphology representation on all length scales from GF to high-level ST.

## 4. A Novel Multi-Scale Morphology Descriptor

To effectively eliminate the inter-scale effect on representing particle morphology at different RLS and to reflect the surface roughness heterogeneity, a novel spherical harmonic-based multi-scale roughness descriptor is proposed in this paper.

### 4.1. Definition

**Figure 5:**
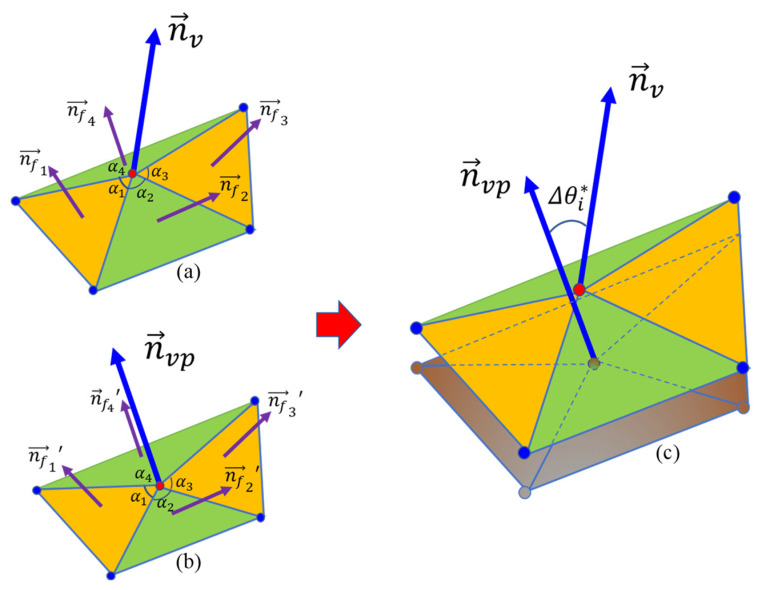
Angle difference between two sub-surfaces with different RLS: (**a**) normal vector for target sub-surface point; (**b**) normal vector for the point with same polar radius direction on previous sub-surface; (**c**) angle difference.

For any point on a given particle surface, its normal vectors on reconstructed sub-surfaces with different RLS can be calculated. Then, the angle difference of normal vectors on decomposed sub-surfaces at target and preceding RLS can reflect the morphology variation at this target RLS. For example, the angle difference between normal vectors of sub-surfaces at LR and 1L-ST can depict the morphology variation of 1L-ST at a given surface point. The calculation of this angle difference at a given point, as illustrated in Figure 5, is given by Equation (6).

(6) $$\Delta \theta_{iv}^{*}=arccos({\vec{n}}_{v},{\vec{n}}_{vp}),$$

where ${\vec{n}}_{v}$ is the normal vector at the point on the target sub-surface and ${\vec{n}}_{vp}$ is the normal vector on the preceding sub-surface. ${\vec{n}}_{v}$ and ${\vec{n}}_{vp}$ can be determined based on the normal vectors of all the triangulated faces sharing the same vertex (i.e., the target point) on the sub-surfaces as follows:

(7) $${\vec{n}}_{v}=norm(\sum_{i}^{k}\alpha_{i}\times {\vec{n}}_{fi}),$$

where ${\vec{n}}_{fi}$ is the normal vector of face *i*, *α_i_* is the angle-related weight for this face *i* (i.e., the angle of the vertex on face *i*), and *k* is the number of faces that share the vertex (*k* = 4 in this case).

Therefore, based on the angle difference $\Delta \theta_{i}^{*}$ between normal vectors on two sub-surfaces, the descriptor *R_inc_* can depict the incremental morphology variation (IMV) and is defined as follows:

(8) Rinc=∑iNS(Δθi*)nS×π2 (Δθi*≥Δθmin*),

where *N_S_* is the number of vertices after filtering, *n_S_* is the number of vertices on the target surface region, and $\Delta \theta_{min}^{*}$ is the filter threshold based on the instrumental resolution (i.e., 6.5 μm in this study) to eliminate the non-physical angle differences introduced by the algorithm, as given in Equation (9).

(9) $$\Delta \theta_{min}^{*}=2\times arcsin(\frac{\sqrt{2}\times P_{Lmin}}{2\times r_{i}}),$$

where *P_Lmin_* is the minimum pixel length (6.5 μm in this study), and *r_i_* is the polar radius of the target point. The value of *R_inc_* varies from 0 to 1, and a larger value denotes greater morphology variation. Specifically, 0 denotes that no IMV occurs at the corresponding RLS, and 1 depicts an overall angle variation of π/2 for all the surface points, which, however, cannot be reached for LBS particles due to the surface continuity.

### 4.2. Variation of R_inc_ with SH Decomposition

**Figure 6:**
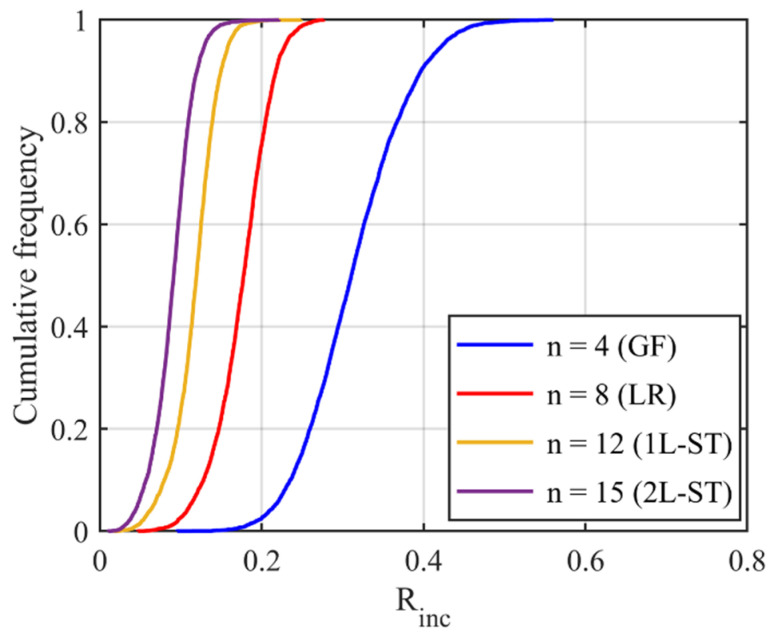
Cumulative distribution of *R_inc_* at different RLS for 4155 LBS particles.

Since *R_inc_* is estimated by the angle difference between normal vectors of the target sub-surface and the corresponding preceding sub-surface, it can be utilized to depict the IMV of a given particle at each RLS and, hence, can act as a morphological gene for the given particle. Different parts of this gene represent morphology variation at different RLS. Table 2 illustrates the variation of *R_inc_* with SH decomposition for a sphere, a cube, and two LBS particles. For a sphere, *R_inc_* is equal to 0 at all RLS because no IMV occurs across different RLS. Unlike the sphere, *R_inc_* values for the cube and two particles show IMV at all RLS. Since *R_inc_* at SH frequency *n* = 4 shows the morphology variation from a sphere to sub-surface of *n* = 4, it can be utilized to reflect the IMV of GF. From Table 2, Particle 0036 is more visually irregular than Particle 0006, and it shows a larger *R_inc_* at *n* = 4 which means a larger IMV needed from a sphere to the target GF. Therefore, *R_inc_* at *n* = 4 can reflect the irregularity of GF for a given particle. Specifically, the cube has large corners which produce large angle differences and, hence, the cube has an overall larger *R_inc_* than these two LBS particles. Moreover, *R_inc_* at *n* = 8, *n* = 12, and *n* = 15 depict the IMV of LR, 1L-ST, and 2L-ST, respectively. Figure 6 illustrates the cumulative distribution of *R_inc_* at different RLS for all the 4155 LBS particles. By using *R_inc_*, each part on the morphological gene corresponds to different target RLS with a uniform format of definition (Equation (8)). From the figure, the IMV at different RLS decreases with the increasing SH frequency, meaning that the IMV at small RLS is lower than that at large RLS, while the overall surface is still getting rougher with decreasing RLS. Due to the limitation of CT resolution, the discussion of *R_inc_* is limited to *n* = 15, i.e., the 2L-ST. However, the *R_inc_* proposed can be utilized to represent IMV at any smaller RLS, as long as particle data provided can meet that resolution. 

### 4.3. Estimating R_inc_ Using Artificial Neural Network (ANN)

To verify the elimination of inter-scale effects, ANN is introduced to correlate *R_inc_* with the SH-based invariants, based on which the mean impact value (MIV) is then calculated. MIV was first proposed by Dombi et al. [50] to reflect the variation of weighted matrix in ANN. The MIV of *R_inc_* at different RLS can reflect the contribution of morphology variation at each RLS to the estimation of *R_inc_* and, hence, can be utilized to verify the effective elimination of inter-scale effects by *R_inc_*. To calculate the MIV of *R_inc_*, an ANN model is established with Levenberg–Marquardt (LM) algorithm [51]. The input and output parameters are the second-order norms of SH frequencies of 1 to 15 and *R_inc_* at different RLS, respectively. The size of the hidden layer is set to be two-thirds of the sum of input and output parameters [52] to accelerate the training and avoid over-fitting. Table 3 depicts the training performance of ANN with mean squared error (MSE) and regression *R* value. As can be seen from the table, all MSEs are extremely close to 0 while the *R* values are greater than 0.97, which reflect the feasibility and accuracy of the ANN model established. In addition, another ANN model is trained with the same input parameters as but different V/S ratio to the output parameter. Since V/S ratio is one of the overall shape parameters that can be independently influenced by morphology information at all RLS, comparisons with MIV results of V/S ratio can further verify the elimination of inter-scale effects.

Based on the ANN model established, each item of the input second-order norms is increased and decreased by 10% to obtain two outputs, and this procedure is repeated for all the samples. The difference between the two output datasets obtained is the impact value (IV). Then, the MIV is calculated by averaging the IV for all samples, as expressed in Equation (10). The absolute MIV represents the relative significance of independent variables with respect to dependent variables, and a greater MIV value reflects a higher influence.

(10) $$MIV_{Xi}=\;\frac{1}{n}\left|\sum_{j}^{n}\frac{Y(X_{i}\times 110\%)-Y(X_{i}\times 90\%)}{Y(X)}\right|,$$

where *X_i_* *× 110%* means the *i-*th item of input data increases by 10% while the others remain unchanged, is the output by ANN, and *n* is the number of samples. 

**Figure 7:**
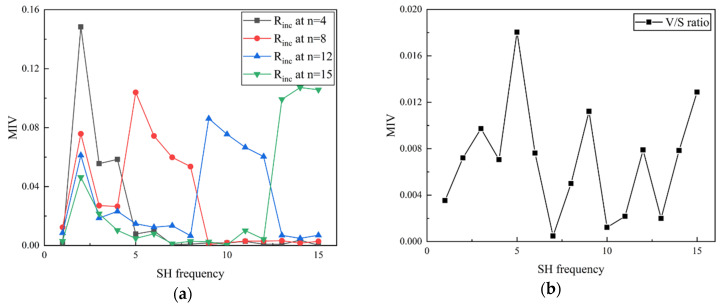
Mean impact va;ue (MIV) results: (**a**) *R_inc_* at different RLS; (**b**) V/S ratio.

Figure 7 shows the MIV results of *R_inc_* and V/S ratio. Solid lines in Figure 7a illustrate the *R_inc_* at different RLS from GF (*n* = 4) to 2L-ST (*n* = 15). The abscissa denotes the SH frequencies which correspond to different RLS, while the ordinate depicts the MIV that can show the significance of each SH frequency in the estimation of *R_inc_*. As can be seen from the figure, *R_inc_* at different RLS shows a generally higher level of sensitivity to the corresponding RLS without inter-scale effects from other RLS. For example, *R_inc_* at *n* = 4, based on the angle difference between normal vectors of sub-surfaces at *n* = 0 and *n* = 4, is highly sensitive to the SH frequency range from *n* = 2 to *n* = 4 and, thus, dominates the GF of the given particle. Similarly, *R_inc_* at *n* = 8 dominates the LR; *R_inc_* at *n* = 12 and *n* = 15 dominate the 1L-ST and 2L-ST, respectively. Furthermore, it is found that the estimation of *R_inc_* will be slightly affected by GF, and this influence becomes lower for the reconstructed particle surface with a higher *n* value. Since, for a given sand particle, the GF is the same for all reconstructed particles with varying *n* values, the GF-related effects can be ignored. The MIVs of V/S ratio in Figure 7b, on the other hand, show an overall low sensitivity without any dominant RLS, which means that morphology information on all RLS can contribute to the evaluation of V/S ratio with similar weights. Therefore, by reflecting the incremental morphology variation at the target RLS without inter-scale effects from other RLS, the *R_inc_* proposed is very suitable for acting as a morphological gene to represent the multi-scale feature of particle morphology.

### 4.4. Variation of R_inc_ against Incremental Surface Area

It is interesting to examine the influence of IMV represented by *R_inc_* on the increase of particle surface area. The rate of the increase of surface area at the target RLS for a given particle can be expressed as follows:

(11) $$R_{SA}\;=\;\frac{S_{Av}-S_{Ap}}{S_{Ap}},$$

where *S_Av_* and *S_Ap_* are the surface area of particle at the target RLS and the preceding RLS, respectively. 

**Figure 8:**
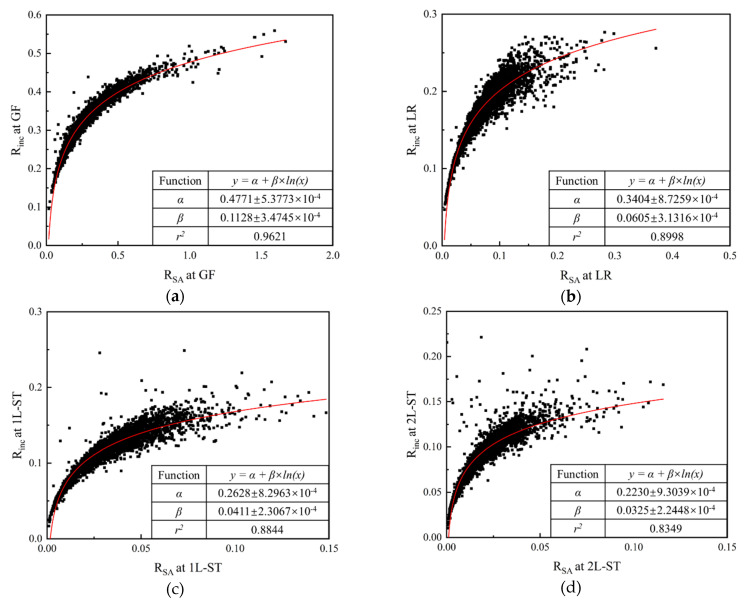
Relationship of *R_inc_* and *R_SA_* at different RLS: (**a**) general form (GF); (**b**) local roundness (LR); (**c**) first-level surface texture (1L-ST); (**d**) second-level surface texture (2L-ST).

Figure 8 depicts the relationship of *R_inc_* and *R_SA_* at different RLS. It can be seen that *R_inc_* vs. *R_SA_* shows a similar pattern of positive correlation for all RLS, and the degree of data scatter actually decreases with the decreasing RLS (note the different scales adopted in different sub-figures of Figure 8). This result clearly suggests that the IMV represented by *R_inc_* is the cause for the increase of particle surface area. The correlations in Figure 8 can be fitted using Equation (12).

(12) $$R_{inc}\;=\;\alpha \;+\;\beta \times ln(R_{SA}),$$

where *α* and *β* are scale-related parameters, and their values for different RLS are listed in the table of the corresponding figure. The correlation indices *r^2^* for all RLS are larger than 0.83, which reflects the feasibility of Equation (12).

### 4.5. Variation of R_inc_ against Traditional Descriptors at Target RLS

**Figure 9:**
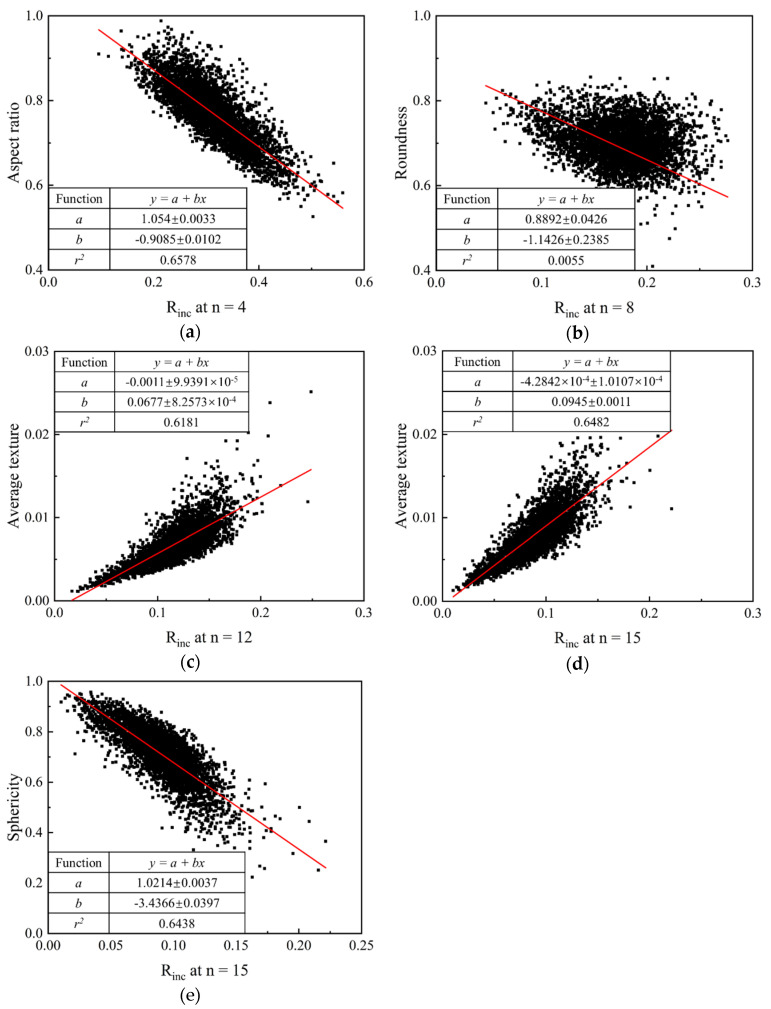
Relationship of *R_inc_* and traditional descriptors at target RLS: (**a**) aspect ratio; (**b**) roundness; (**c**) average texture of 1L-ST; (**d**) average texture of 2L-ST; (**e**) sphericity.

To help better understand the descriptor *R_inc_* proposed in this paper, we correlate *R_inc_* with traditional descriptors at different target RLS, as shown in Figure 9. As can be seen from the figure, *R_inc_* shows a negative correlation with aspect ratio and sphericity while a positive one with average texture at the corresponding RLS. Specifically, *R_inc_* at *n* = 4, as discussed above, reflects the IMV of GF, and a larger *R_inc_* at *n* = 4 leads to a smaller aspect ratio. The intercept on the ordinate axis is around 1 which means that *R_inc_* at *n* = 4 equal to 0 depicts a sphere. In addition, sphericity shows a similar variation trend with aspect ratio. This intercept on the ordinate axis is also around 1 which depicts that, for a sphere, *R_inc_* = 0 at all RLS. Average textures of both 1L-ST and 2L-ST, on the other hand, illustrate an opposite variation trend, i.e., increasing with increasing *R_inc_* at the corresponding RLS. Furthermore, from Figure 9b, roundness is poorly correlated to *R_inc_* at *n* = 8. This is because the estimation of roundness is based on the curvature radius of all corners, as well as the maximum inscribed circle [20,53,54] and, hence, roundness focuses on the convex part for a given particle. Unlike roundness, *R_inc_* at *n* = 8 reflects the IMV from GF to LR, and it contains morphology variation of both convex and concave parts for a given particle.

### 4.6. Surface Roughness Heterogeneity by R_inc_

Obviously, *R_inc_* (Equation (8)) can be utilized to evaluate morphology variation for any local region of the particle surface at a target RLS. This value represents the IMV at an individual point *i* when *n_S_* = 1, while it represents the IMV of the whole particle when the target surface region is equal to the entire particle surface. 

**Figure 10:**
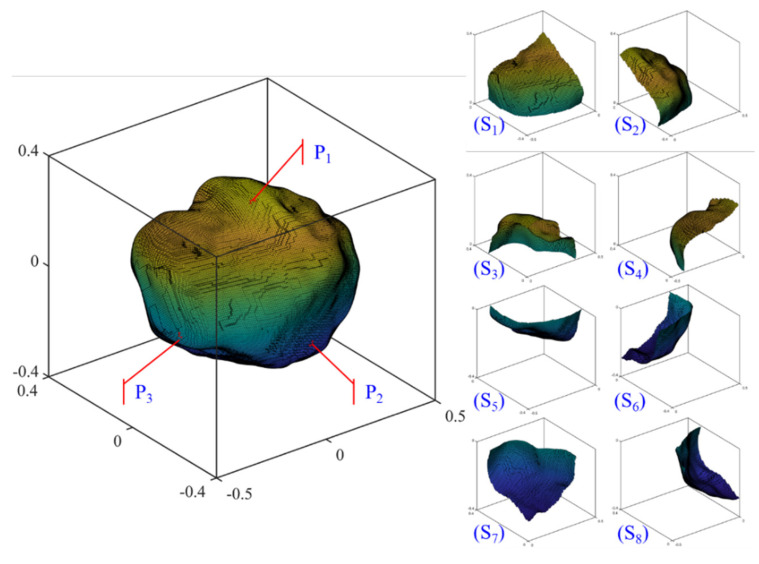
Visualization of local points and surfaces.

Three local points P1, P2, and P3 are selected randomly from a given particle surface, and eight parts of the surface are obtained by dividing the given particle with the three Cartesian coordination planes across the particle center (i.e., the XOY, YOZ, and XOZ planes), as shown in Figure 10, are used to illustrate the surface roughness heterogeneity. Table 4 lists the surface roughness heterogeneity quantified by different *R_inc_* values at different RLS. For the whole particle, the *R_inc_* values are 0.2003 for GF, 0.1303 for LR, and 0.1051 and 0.0658 for 1L-ST and 2L-ST. However, the three local points show different *R_inc_* values at all RLS, reflecting the roughness heterogeneity of particle morphology. A similar phenomenon can be seen from the *R_inc_* of all the local surfaces at different RLS. Specifically, the local surface S1 shows a higher *R_inc_* than S2 for the GF, LR, and 2L-ST, but a lower value for the 1L-ST. This observation implies that the *R_inc_* at different RLS are independent of each other, meaning that a given surface may be smooth at one RLS but rough at other RLS.

## 5. Conclusions

A combined X-ray *μ*CT and SHA technique was utilized to decompose the given particle surface into a series of sub-surfaces at different RLS. A total number of 4155 LBS particles were used to create a large dataset of particle surfaces with different target RLS. 

Four groups of traditional morphology descriptors were selected at different RLS to investigate the effect of SH decomposition and the inter-scale effect. It was found that the inter-scale effect, either from large scale or from small scale, affects the estimation of particle morphology at all RLS.

To effectively eliminate the inter-scale effect and precisely represent the morphology variation of a given particle at different RLS, a novel spherical harmonic-based multi-scale morphology descriptor *R_inc_* was proposed. It is concluded that, firstly, by investigating the variation of *R_inc_* against RLS, the particle surface is, in general, rougher with decreasing RLS, but the IMV at each RLS shows a decreasing trend. Secondly, ANN-based MIVs of *R_inc_* at different RLS are calculated and the results show a general high level of sensitivity to the corresponding RLS without inter-scale effects from other RLS, which proves the effective elimination of the inter-scale effect. Thirdly, by introducing the increase rate of surface area *R_SA_*, the *R_inc_* proposed shows a positive correlation with *R_SA_* at all RLS, and it can be expressed by $R_{inc}\;=\;\alpha \;+\;\beta \times ln(R_{SA})$, where *α* and *β* are scale-related parameters. Fourthly, by correlating *R_inc_* with traditional descriptors at target RLS, *R_inc_* at the corresponding RLS can be utilized as an alternative method to quantify the irregularity and roughness for a given particle. Lastly, the surface roughness heterogeneity was investigated using *R_inc_*, and it was found that the surface morphology of a given particle shows different IMV in different sections, as well as different variation trends at different RLS.

Therefore, with the merits of (1) having a uniform format of definition across all RLS, (2) effectively eliminating the inter-scale effects, and (3) reflecting the surface roughness heterogeneity, the *R_inc_* proposed in this paper is very suitable for acting as a morphological gene to represent the multi-scale feature of particle morphology.

## Figures and Tables

**Table 1:** Definitions of morphology descriptors.

Group	Descriptor	Formula	Definition	References
General form	Elongation	$E=\frac{I}{L}$	Ratio of the second principal dimension (*I*) over the first principal dimension (*L*)	[21,44]
	Flatness	$F=\frac{S}{I}$	Ratio of the third principal dimension (*S*) over the second principal dimension	
	Aspect ratio	$AR=\frac{E+F}{2}$	The mean value of elongation and flatness	
Local roundness	Roundness	$R=\;\frac{\sum R_{c}}{n_{c}\times R_{insc}}$	Ratio of all corner curvature radii ($R_{c}$) to the largest inscribed sphere radius (*R_insc_*)	[20,45]
Surface texture	Average texture	$R_{a}=\frac{\sum \left|Z_{i}\right|}{n}$	The arithmetic average of the target surface departure from the mean surface	[46]
Overall shape parameter	Sphericity	$S\;=\;\sqrt[{3}]{\frac{36\pi V^{2}}{S_{A}}}$	Ratio of the surface area (*S_A_*) of a sphere with the same volume (*V*) as the given particle to surface area of this particle	[20,47]
	Ratio of volume to surface area	$\frac{V}{S_{A}}$	Ratio of particle volume to particle surface area	[48]
	Convexity	$C_{x}=\frac{V}{V_{CH}}$	Ratio of particle volume over its convex hull volume (*V_CH_*)	[49]

**Table 2:** Variation of *R_inc_* with SH decomposition.

**Particle**	*n* = 4	*n* = 8	*n* = 12	*n* = 15
**Sphere**	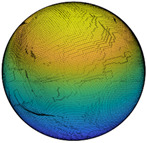 *R_inc_* = 0	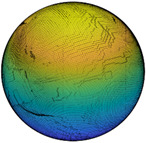 *R_inc_* = 0	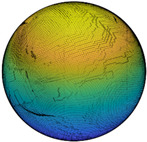 *R_inc_* = 0	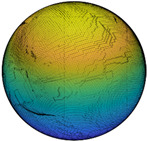 *R_inc_* = 0
**Cube**	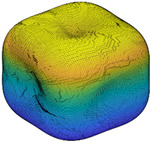 *R_inc_* = 0.2877	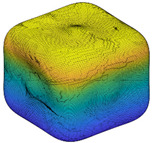 *R_inc_* = 0.1608	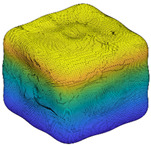 *R_inc_* = 0.1308	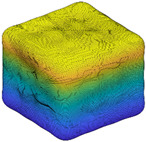 *R_inc_* = 0.0738
**Particle 0006**	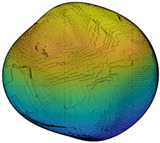 *R_inc_* = 0.2003	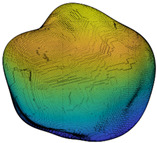 *R_inc_* = 0.1303	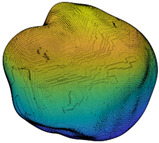 *R_inc_* = 0.1051	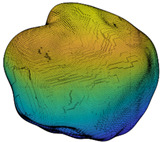 *R_inc_* = 0.0658
**Particle 0036**	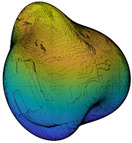 *R_inc_* = 0.2839	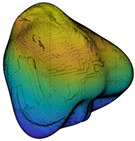 *R_inc_* = 0.1500	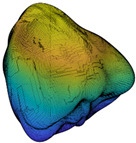 *R_inc_* = 0.1076	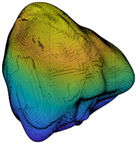 *R_inc_* = 0.0748

**Table 3:** Training performance of artificial neural network (ANN). MSE—mean squared error.

Data Set	Samples	MSE (×10^−4^)	*R*
Training	3323	5.0937	0.9715
Validation	416	4.6397	0.9746
Testing	416	5.2094	0.9709

**Table 4:** Surface roughness anisotropy by *R_inc_* for a given particle.

		*R_inc_ n* = 4 (GF)	*R_inc_ n* = 8 (LR)	*R_inc_ n* = 12 (1L-ST)	*R_inc_ n* = 15 (2L-ST)
**Whole Particle**		0.2003	0.1303	0.1051	0.0658
Local points	P1	0.1522	0.0878	0.1425	0.1251
	P2	0.1683	0.1852	0.1444	0.1093
	P3	0.3550	0	0.0526	0.1033
Local surfaces	S1	0.2051	0.1163	0.0964	0.0806
	S2	0.1803	0.1079	0.1063	0.0692
	S3	0.1713	0.1203	0.1580	0.0726
	S4	0.1786	0.1235	0.0725	0.0625
	S5	0.2988	0.1336	0.0821	0.0675
	S6	0.1954	0.1171	0.0945	0.0583
	S7	0.1427	0.1683	0.1451	0.0686
	S8	0.2416	0.1567	0.0838	0.0477

## Abbreviations

ANN	Artificial neural network
CT	Computed tomography
GF	General form
IMV	Incremental morphology variation
LBS	Leighton Buzzard Sand
LOESS	locally weighted regression smoothing
LR	Local roundness
MIV	Mean impact value
RLS	Relative length scale
SH	Spherical harmonics
SHA	Spherical harmonic analysis
SSRF	Shanghai Synchrotron Radiation Facility
ST	Surface texture
V/S ratio	Ratio of volume to surface area
*μ*CT	Micro-tomography
1L-ST	First-level surface texture
2L-ST	Second-level surface texture
3D	Three-dimensional
